# Comparison of Insulin Lispro Protamine Suspension with NPH Insulin in Pregnant Women with Type 2 and Gestational Diabetes Mellitus: Maternal and Perinatal Outcomes

**DOI:** 10.1155/2013/151975

**Published:** 2013-06-10

**Authors:** Antonietta Colatrella, Natalia Visalli, Santina Abbruzzese, Sergio Leotta, Marzia Bongiovanni, Angela Napoli

**Affiliations:** ^1^Department of Clinical and Molecular Medicine, S. Andrea Hospital, Faculty of Medicine and Psychology, Sapienza University, Via di Grottarossa 1035–1039, 00189 Rome, Italy; ^2^Unit of Dietology, Diabetology and Metabolic Diseases, Sandro Pertini Hospital, Via dei Monti Tiburtini 385, 00157 Rome, Italy

## Abstract

Insulin therapy is still the gold standard in diabetic pregnancy. Insulin lispro protamine suspension is an available basal insulin analogue. *Aim*. To study pregnancy outcomes of women with type 2 and gestational diabetes mellitus when insulin lispro protamine suspension or human NPH insulin was added to medical nutrition therapy and/or short-acting insulin. *Methods*. In this retrospective study, for maternal outcome we recorded time and mode of delivery, hypertension, glycaemic control (fasting blood glucose and HbA1c), hypoglycemias, weight increase, and insulin need. For neonatal outcome birth weight and weight class, congenital malformations was recorded and main neonatal complications. Two-tail Student's *t*-test and chi-square test were performed when applicable; significant *P* < 0.05. *Results*. Eighty-nine pregnant women (25 with type 2 diabetes and 64 with gestational diabetes mellitus; 53 under insulin lispro protamine suspension and 36 under human NPH insulin) were recruited. Maternal and neonatal outcomes were quite similar between the two therapeutic approaches; however, insulin need was higher in NPH. At the end of pregnancy, eight women with gestational diabetes continued to use only basal insulin analogue. *Conclusions*. Pregnancy outcome in type 2 and gestational diabetes mellitus with insulin lispro protamine suspension was similar to that with NPH insulin, except for a lower insulin requirement.

## 1. Introduction 

Insulin therapy is still the gold standard in the treatment of diabetes in pregnancy when medical nutrition therapy (MNT) and lifestyle cannot reach and maintain the metabolic targets [1, 2].

Studies using lispro and aspart in pregnancy have shown that both rapid-acting insulin analogues are safe and effective in reducing the postprandial glycaemia [3–8]. 

However, human neutral protamine Hagedorn (NPH) insulin still remains the primary basal insulin choice [9–13], even though, recently, a randomized controlled trial demonstrated noninferiority of detemir versus NPH insulin in 310 pregnant women with type 1 diabetes [14]. 

Insulin lispro protamine suspension (ILPS), formulated by cocrystallizing insulin lispro with protamine, is a basal insulin analogue with pharmacokinetics and glucodynamics comparable to those of NPH insulin [15]. Current evidence suggests that ILPS may represent a valuable option in the management of diabetic patients, primarily those with type 2 diabetes, requiring insulin treatment regimens [16]. There are no studies about ILPS use during pregnancy, during which the continuous adjustment of the hormonal pattern causes several metabolic and circulatory changes in the mother's body to accommodate fetal needs [17], so its metabolic effectiveness in pregnancy has not yet been demonstrated. To this aim, we retrospectively studied pregnancies in women with type 2 and gestational diabetes mellitus (GDM) when ILPS or NPH insulin was added to MNT and/or rapid insulin analogues. 

Our primary objective was to compare the main maternal outcomes (mode and time of delivery, preterm delivery, and hypertensive disorders) and perinatal outcomes (birth weight and weight class, congenital malformations, neonatal hypoglycaemia, and other perinatal morbidities). As a secondary objective, we evaluated other clinical and glycaemic outcomes (fasting blood glucose and HBA1C, hypoglycemic episodes, insulin need, and weight gain) between the two therapeutic approaches (ILPS or NPH insulin). 

## 2. Patients and Methods

This is a multicentre retrospective observational study of a cohort of pregnant women affected by type 2 or gestational diabetes mellitus (GDM) which was not being effectively controlled with MNT and/or rapid insulin analogues, who were additionally treated with an intermediate-acting insulin (ILPS or NPH). 

All women were recruited consecutively from January 2008 to August 2010 in two hospitals located in Rome (S. Andrea and Sandro Pertini).

## 3. Study Protocol

Pregnancy dating, based on menstrual history and physical examinations, was definitely confirmed by an early ultrasound examination before the 16th week of gestation. 

GDM was diagnosed between the 24th and 28th weeks of gestation with an (75 or 100 g) oral glucose tolerance test (OGTT); results were interpreted according to the Carpenter and Coustan criteria [18] and the recommendations of the 4th International Workshop Conference on Gestational Diabetes Mellitus [19]. In cases with one or more GDM risk factors (family history of type 2 diabetes, history of GDM and/or impaired glucose tolerance, obesity, and glycosuria), diagnosis was done earlier, as soon as it was feasible [19] to do so. All women with type 2 diabetes were treated with diet and/or oral hypoglycemic agents before pregnancy. At conception, 8 of these women were treated with metformin, 1 with metformin + sulphonylurea, 2 with sulphonylurea, 2 with repaglinide, 2 with metformin + repaglinide, 1 with metformin + rosiglitazone, 1 with insulin, and 8 with diet only. At the first visit, oral hypoglycaemic agents were shifted to diet only or diet plus insulin.

Glycaemic control was obtained when the following standardized goals were reached: fasting and preprandial ≤95 mg/dL (5.3 mmol/L) and 1 h after-meal ≤140 mg/dL (7.8 mmol/L) [20]. 

The individualized MNT was prescribed according to the patient's own preferences (ethnic, cultural, financial, etc.), physical activity level, gestational age, and prepregnancy BMI group with a distribution of carbohydrate intake of 45–50%, 30–35% of lipids, 20% of protein, and 28 g/day of fibers [9]. When MNT was not sufficient to control postprandial hyperglycemia, short-acting insulin analogues such as aspart or lispro were injected before meals; when MNT was not sufficient to control fasting hyperglycemia, basal insulins such as ILPS or NPH were prescribed at bed time; in those few cases in which preprandial glucose values were higher than targets, ILPS or NPH was added before breakfast as well [9]. 

Taking into account the safety of lispro, ILPS and NPH insulins were autonomously prescribed, often on the basis of their different commercial availability in the area where the women came from, that is, the consequence of some brands' policy which is removing NPH insulin from the Italian market.

At the moment of enrollment (coinciding with the introduction of basal insulin), within the ILPS group, 36 patients were already being treated with rapid analogue, while 17 were being treated only with diet; within the NPH group, all the patients were already being treated with rapid analogue.

Patients were taught to self-monitor their plasma glucose levels 4–6 times a day, using the same type of glucometer, given to them by our staff. All data was recorded in a diary kept by the patients at each control visit. Maternal glycohemoglobin (HbA1c) was checked every 4–6 weeks.

The diabetic women were visited at regular intervals (1-2 weeks). At each visit, home capillary blood glucose profiles, insulin requirement and adjustments, hypoglycemic episodes, and body weight were recorded. Capillary blood glucose profiles during the previous 1 or 2 weeks were recorded as mean values ± standard deviation (SD).

Regarding the maternal outcomes, we recorded time and mode of delivery, hypertensive disorders, glycaemic control (as fasting capillary blood glucose, FCBG, and HbA1c), hypoglycemic episodes, weight increase, and insulin need.

Preterm deliveries were those occurring before the 37th gestational week. 

Hypertension was defined according to the National High Blood Pressure Education Program Working Group on High Blood Pressure in Pregnancy [21] and classified as follows: chronic hypertension as hypertension before the 20th week of gestation (CR); gestational hypertension (PIH) as hypertension after the 20th week of gestation; preeclampsia (PE) as gestational hypertension + proteinuria (0.3 g/24 h). We considered hypertensive disorders as CR+PIH+PE.

The degree of hypoglycaemic episodes was graded as follows: mild if slight symptoms spontaneously were resolved; moderate if symptoms resolved by taking oral carbohydrate; severe if symptoms were resolved requiring assistance from another person; serious if they required admittance to hospital. 

Prepregnancy BMI was calculated according to our patients' height/reported pregestational weight (kg)^2^ [22]. Insulin need (as units/kg/day) was calculated as total daily insulin doses (rapid + intermediate-acting insulin divided body weight), daily rapid insulin (daily rapid insulin/body weight), and daily intermediate-acting insulin (daily ILPS or NPH insulin/body weight) at the last visit before delivery; daily rapid insulin doses were also calculated before the introduction of ILPS or NPH (enrollment).

For neonatal outcomes, we noted the length and the weight at birth, APGAR score at 5 minutes, congenital malformations, hypoglycemia, hyperbilirubinemia, other neonatal morbidity (as obstetric trauma, respiratory disorders, and need for intensive cure—NICU—), stillbirths, and neonatal mortality. 

We calculated the ponderal index (PI) as the ratio of weight to length cubed (g/cm^3^), considering a PI higher than 2.85 g/cm^3^ as excessive [23]. Babies were defined large for gestational age (LGA) if their birth weight was above the 97th percentile and small for gestational age (SGA) if their birth weight was below the 3rd percentile, based on standard growth and development tables for the Italian population [24]. Malformations were classified according to the EUROCAT (http://www.eurocat-network.eu/) and fetal morbidity to the Obstetrical Quality Indicator [25]. Stillbirths were children born dead beyond 180 days of pregnancy. Neonatal mortality was the rate of deaths before the 28th day of life.

Women gave their written consent for the anonymous use of their clinical data at the first visit, as previously approved by our ethics committee.

## 4. Statistics

All data was presented as means ± standard deviation (SD) for continuous variables and as percentages for categorical variables. Data was processed with the Apple software program (Stat View). Two-tail unpaired and paired Student's *t*-tests were used when applicable to compare the pairs of means or longitudinal values. Chi-square (*χ* ^2^), as nonparametric test, was performed to compare percentages. *P-*values of <0.05 were considered significant.

## 5. Results

Eighty-nine diabetic pregnant women treated with a basal insulin (ILPS or NPH) were consecutively recruited from January 2008 to August 2010 in two diabetes units in Rome (S. Andrea and Sandro Pertini). 

Twenty-five of them were affected by type 2 diabetes and sixty-four by GDM. ILPS (ILPS group or ILPSg, *n* = 53) or NPH (NPH group or NPHg, *n* = 36) was introduced in addition to the current MNT ± rapid insulin analogues therapy when fasting plasma glucose was above the ADA goal.

Because there was a higher number of women with type 2 diabetes in NPHg (*χ* ^2^ 0.0002), we separated those with type 2 diabetes from those with GDM; no significant differences of main clinical characteristics between treatment groups (ILPSg versus NPHg) at baseline were reported (see Table 1).

The duration of type 2 diabetes was 7.0 ± 4.8 years with no differences between groups (ILPSg 8.0 ± 5.2 versus NPHg 6.6 ± 4.7 yrs, ns); GDM was diagnosed at 21.9 ± 7.3 weeks with no differences between ILPSg and NPHg (respectively, 22.6 ± 7.4 versus 20.3 ± 6.7 wks, ns). At enrollment, in the ILPSg, 17 women (16 with GDM and one with type 2 diabetes) needed only a basal insulin (*χ* ^2^ 0.002) and 36 had already been treated by short-acting insulin analogues, with no difference between ILPS and NPH groups (Table 1). At the end of pregnancy; eight of these patients (all with GDM) continued to use ILPS only, with an insulin need of 0.06 ± 0.02 IU/kg/day (*P* < 0.0001). 

In 92.4% of ILPSg versus 88.9% of NPHg (ns), glycaemic control was reached with one basal-acting insulin ± rapid analogues before meals. Among type 2 diabetic pregnancies, only two of 7 (28.6%) women being treated with ILPS and three of 18 (16.7%) being treated with NPH needed two basal insulins plus three short-acting insulin injections; the same therapy was used in two of 46 GDM (4.3%) of the ILPSg and one of 18 GDM (5.5%) of the NPHg.

As short-acting analogue, lispro was used in 51% of the ILPSg and in 50% the NPHg (ns).

## 6. Primary Endpoints

Maternal outcome was similar between ILPSg and NPHg in terms of mode and time of delivery (Table 2). The rate of hypertension, which was higher in all of NPHg, was found to be similar when patients with type 2 diabetes were split from those with GDM (hypertensive disorders in type 2 diabetes: 42.8% in ILPSg versus 44.4% in NPHg, ns; GDM: 17.4 ILPSg versus 33.3% NPHg, ns).

Neonatal outcomes did not statistically differ (Table 2), except when considering excessive ponderal index. Of three newborns whose ponderal index was >2.85 g/cm^3^, two were delivered by mothers affected by GDM and one by type 2 diabetes. Two of these mothers were treated with aspart and one with lispro as short-acting analogue. All these women had used NPH as basal insulin. Those women whose pregestational BMI was lower showed a lower weight gain, with higher capillary blood glucose levels and a higher insulin need. However, all these parameters did not reach significant levels.

Three newborns reported minor congenital malformations (two in the ILPSg and one in the NPHg, ns) consisting in heart defects (patent or persistent foramen ovale), not requiring surgery.

No newborn was SGA. There were no stillbirths or neonatal deaths nor neonatal complications needing intensive care (data not shown in table).

## 7. Secondary Endpoints

Fasting capillary blood glucose values and the number/severity of hypoglycaemic episodes were available for 58 women only (30 in the ILPSg and 28 in the NPHg).

FCBG were not different throughout pregnancy (Table 3). As we considered FCBG < 95 mg/dL, this cutoff was obtained in 66.7% of the ILPSg versus 66.8% of the NPHg (ns) at the end of pregnancy. 

Both groups of patients reported only mild and/or moderate hypoglycaemias, with no severe and serious episodes. The number of reported events was similar for both therapies (ILPSg 0.3 ± 0.3 versus NPHg 0.3 ± 0.7, ns; type 2 ILPSg 0.2 ± 0.5 versus NPHg 0.5 ± 1.0, ns; GDM ILPSg 0.1 ± 0.3 versus NPHg 0.1 ± 0.2, ns).

Weight gain did not differ between the two treatment-groups, in type 2 diabetic pregnant women as well as in those with GDM (Table 3).

Insulin need was higher in the NPHg either as total daily insulin in both types of diabetes or as rapid-acting analogue in type 2 diabetic women only (Table 4). 

## 8. Discussion 

Taking into account the physiological changes of glycemic profiles in pregnancy [26] and the consolidated results [27] that adjustment of postprandial, rather than preprandial, blood glucose values improve pregnancy outcomes, it is generally known that insulin analogues may produce better glycaemic control with less hypoglycemia risk compared with the use of human insulin (level of evidence: E) [9]. However, only lispro and aspart are currently used in pregnancy, pending clinical trials definitively proving safety and efficacy of other analogues (such as glulisine and glargine) [9]. Recently, a randomized controlled trial in 310 pregnant women with type 1 diabetes demonstrated noninferiority of detemir versus NPH insulin in terms of maternal efficacy (HbA1c) and safety (hypoglycemia) [14]. So, the FDA has reclassified insulin detemir from pregnancy category C to pregnancy category B (http://www.fda.gov/drugs/drugsafety/). 

Gestational diabetes is a metabolically heterogeneous disorder; therefore, when high fasting blood glucose values are found, treatment with a basal insulin is compulsory. ILPS could be an “on label” alternative therapeutic option to NPH insulin. To this aim, we retrospectively evaluated pregnancy outcome in women with GDM or type 2 diabetes mellitus treated with ILPS or NPH insulin. 

As the distribution of the two types of diabetes was different in the two treatment approaches, we divided the results into subgroups. Not surprisingly, the earlier gestational age at enrollment of type 2 diabetic patients could explain the difference in weight increase from prepregnancy when compared to those with GDM, even though this difference disappeared by the end of pregnancy. However, as GDM was diagnosed quite early, we cannot exclude the extent to which some of the women classified as GDM were affected by unknown type 2 pregestational diabetes.

Overall, the maternal and neonatal outcomes of diabetic women treated with ILPS for about half of pregnancy were not different from those of women treated with NPH. 

The high prevalence of hypertension found in both types of diabetes, not associated with any of the two basal insulins used, was similar to that reported by other studies [28, 29].

Birth weight, ponderal index, and prevalence of LGA were similar in the two groups. However, an excessive ponderal index, a more precise measure of obesity in pediatrics, was found in three newborns of women with a worse metabolic control and higher insulin requirement, all belonging to the NPHg. There are contrasting results on the birth weight of newborns of diabetic pregnancies under lispro [6, 30, 31]. Our data does not show an increased birth weight in the ILPSg when risk factors are similar (maternal BMI and weight gain, type of diabetes), taking into account that the percentage of aspart users was similar in the two groups. This result is important as an indirect effect of insulin analogues through placental action is hypothesized [30].

Metabolic control was similar and quite satisfactory as fasting blood glucose goals were reached in about two/thirds of each group. Moreover, the reduction of FCBG throughout pregnancy, even if not always significant, is the expression of stabile metabolic control in a time span characterized by a progressive increase of insulin resistance [32].

We cannot draw any conclusion about the influence of either ILPS or NPH on hypoglycemia as the episodes were all mild or moderate and extremely rare.

In fact, although hypoglycemia is reported as a complication in type 1 diabetic women, with a higher incidence in early pregnancy [33, 34], this finding is less frequent in type 2 and gestational diabetes because of the different pathogenesis of the disease. 

The low insulin requirement in both groups can be explained by our patients' BMI which was significantly less than described in other populations [35] as well as by the adherence to MNT, as indirectly shown by the weight gain, and the short duration of disease for type 2 diabetes.

Interestingly, total daily insulin need was higher both in type 2 diabetes and GDM treated with NPH while a higher need for short-acting analogue was found in type 2 diabetic women only. Moreover, eight women with GDM were well controlled by ILPS at bedtime only. These data could be due to a longer duration of glucose-lowering activity of ILPS in this population [16]. 

Indeed, recent comparative studies examining the pharmacokinetic and pharmacodynamic profiles [36] or the efficacy and safety of ILPS with the other two long-acting insulin analogues [16] suggest that ILPS has a similar glycemic control to glargine and detemir in type 2 diabetes mellitus, out of pregnancy.

Today, caution in the use of most insulin analogues during pregnancy [9] is derived from available data that is incomplete and sometimes contradictory [37]. 

The placenta expresses both insulin-like growth factor 1 (IGF-1) and insulin receptors on both the maternal and fetal sides. As pregnancy progresses, insulin receptors are reduced while IGF-1 receptors do not suffer significant changes on the maternal side [38]. Insulin analogues may interact differently with insulin or IGF-1 receptors, activating metabolic and mitogenic pathways in a different way from native insulin [39] and opening the possibility of an indirect effect of maternal insulin on the fetus. Careful attention must be paid to the use of long-acting insulin analogues during pregnancy due to increased mitogenic activity observed *in vitro* with respect to insulin and short-acting analogues [40].

For this reason, the use of ILPS is a viable therapeutic option when pregnant women with type 2 or gestational diabetes need a basal insulin. 

The limitations of this study are based on the retrospective nature as well as the limited size of the study group (mainly, of the type 2 diabetic women). However, despite the limited sample size, differences between ILPS and NPH were large enough to reach statistical significance. 

Thus, the descriptive nature of our study cannot give firm conclusions, but it is more of a basis for hypotheses and new studies about the use of ILPS in pregnancy.

Very recently a retrospective study of pregnant women with type 1 diabetes using either insulin detemir (*n* = 67) or glargine (*n* = 46) demonstrated that pregnancy outcome was comparable, except for a lower prevalence of large for gestational age infants in women on glargine [41].

Larger and prospective studies comparing the three long-acting analogues should be encouraged to evaluate their safety and efficacy in pregnancy.

In conclusion, this retrospective multicenter study demonstrates that pregnancy outcome in women with type 2 and gestational diabetes mellitus treated with insulin lispro protamine suspension was similar to those of those treated with NPH insulin, except for a lower insulin requirement.

## Figures and Tables

**Table 1:** Baseline characteristics of pregnant women receiving ILPS or NPH insulin.

	ILPSg	NPHg	*P*
No.	53	36	
Type of diabetes (T2DM/GDM)	7/46	18/18	0.0002
Caucasian ethnicity (%)	90.6 (48/53)	91.7 (33/36)	ns
Age (yrs)			
Total	34.7 ± 5.8	34.2 ± 4.8	ns
T2DM	36.0 ± 4.3	33.4 ± 5.3	ns
GDM	34.5 ± 6.0	34.9 ± 4.3	ns
Prepregnancy BMI (kg/m^2^)			
Total	28.6 ± 5.7	28.8 ± 7.2	ns
T2DM	30.9 ± 6.0	29.2 ± 7.2	ns
GDM	28.3 ± 5.7	28.4 ± 7.3	ns
Gestational week at 1st visit			
Total	22.2 ± 8.4	14.1 ± 8.9	<0.0001
T2DM	15.4 ± 9.6	9.2 ± 6.6	ns
GDM	23.3 ± 7.8	19.0 ± 8.3	ns
Gestational week at enrollment			
Total	27.8 ± 8.1	20.3 ± 11.1	0.0004
T2DM	15.4 ± 13.2	11.1 ± 7.1	ns
GDM	29.1 ± 6.3	29.5 ± 4.9	ns
Weight increase at enrollment (kg)			
Total	7.7 ± 6.7	4.4 ± 4.4	0.01
T2DM	4.7 ± 6.7	1.7 ± 2.4	ns
GDM	8.2 ± 6.7	7.1 ± 4.3	ns
Preenrollment short-acting insulin dose (units/kg/day)			
Total	0.18 ± 0.10	0.27 ± 0.16	0.01
T2DM	0.22 ± 0.13	0.30 ± 0.18	ns
GDM	0.18 ± 0.09	0.23 ± 0.14	ns

BMI: body mass index; T2DM: type 2 diabetes mellitus; GDM: gestational diabetes mellitus.

**Table 2:** Maternal and fetal outcomes in pregnant women receiving ILPS or NPH insulin.

		ILPSg			NPHg	
Number of subjects	Total	T2DM	GDM	Total	T2DM	GDM
	53	7	46	36	18	18
Maternal outcome						
Gestational week at delivery	38.3 ± 1.4	38.4 ± 2.1	38.3 ± 1.3	38.6 ± 1.0	38.9 ± 0.9	38.4 ± 1.1
Caesarean section (%)	67.9	100	65.2	80.5	100	61.1
Preterm delivery (%)	7.5	0	8.7	2.8	0	5.5
Hypertensive disorders (%)	20.7	42.8	17.4	38.9	44.4*	33.3
Neonatal outcome						
Newborn weight (g)	3328.5 ± 517.1	3104.3 ± 444.7	3361.3 ± 523.5	3376.3 ± 604.2	3338.5 ± 669.3	3409.4 ± 561.2
LGA (%)	15.1	14.3	15.2	22.2	22.2	22.2
PI (g/cm^3^)	2.2 ± 0.3	2.1 ± 0.3	2.2 ± 0.3	2.3 ± 0.3	2.3 ± 0.4	2.3 ± 0.3
PI > 2.85 g/cm^3^ (%)	0	0	0	8.3	5.5	11.1
APGAR at 5′	9.7 ± 0.5	9.8 ± 0.4	9.7 ± 0.5	9.5 ± 0.7	9.4 ± 0.8	9.6 ± 0.5
Congenital malformations (%)	3.8	0	4.3	2.8	0	5.5
Neonatal hypoglycemia (%)	7.5	0	8.7	8.3	11.1	5.5
Hyperbilirubinemia (%)	9.4	0	10.9	8.3	11.1	5.5

T2DM: type 2 diabetes mellitus; GDM: gestational diabetes mellitus; LGA: large for gestational age; PI: ponderal index.

*ILPS versus NPH *P* = 0.02.

**Table 3:** Glycaemic control and weight gain in pregnant women receiving ILPS or NPH insulin.

	ILPSg	No. pt	NPHg	No. pt	*P*
FCBG mg/dL (mmol/L)					
Before enrollment					
Total	100.7 ± 15.1 (5.6 ± 0.8)	21	100.6 ± 17.4 (5.6 ± 1.0)	25	ns
T2DM	110.0 ± 7.8 (6.1 ± 0.4)	4	109.8 ± 15.8 (6.1 ± 0.9)	12	ns
GDM	98.6 ± 15.8 (5.5 ± 0.9)	17	92.2 ± 14.5 (5.1 ± 0.8)	13	ns
At the end of pregnancy					
Total	93.6 ± 13.4** (5.2 ± 0.7)	30	95.7 ± 10.8* (5.3 ± 0.6)	28	ns
T2DM	89.2 ± 12.7* (4.9 ± 0.7)	4	95.5 ± 8.2° (5.3 ± 0.4)	13	ns
GDM	94.3 ± 13.5* (5.2 ± 0.7)	26	95.8 ± 12.8* (5.3 ± 0.7)	15	ns
HbA1c% (mmol/L)					
At the end of pregnancy					
Total	5.4 ± 1.1 (36.0 ± 12.2)	34	5.3 ± 0.7 (34.4 ± 8.1)	33	ns
T2DM	5.8 ± 1.1 (39.6 ± 12.6)	7	5.4 ± 0.7 (36.8 ± 8.2)	18	ns
GDM	5.3 ± 1.1 (35.0 ± 12.1)	27	5.0 ± 0.6 (31.5 ± 7.2)	15	ns
Weight gain (kg)					
At the end of pregnancy					
Total	10.4 ± 6.1	53	9.9 ± 4.2	36	ns
T2DM	12.0 ± 6.1	7	10.8 ± 4.9	18	ns
GDM	10.2 ± 6.1	46	9.1 ± 3.3	18	ns

FCBG: fasting capillary blood glucose; T2DM: type 2 diabetes mellitus; GDM: gestational diabetes mellitus.

Paired *t*-test's *P* between preenrollment versus the end of pregnancy: *ns; **0.02; °0.004.

**Table 4:** Insulin need (units/kg/day) at the end of pregnancy in women receiving ILPS or NPH insulin.

	ILPSg (*n* = 53)	NPHg (*n* = 36)	*P*
Total insulin			
Total	0.36 ± 0.27	0.58 ± 0.20	<0.0001
T2DM	0.50 ± 0.22	0.68 ± 0.18	<0.05
GDM	0.34 ± 0.28	0.48 ± 0.19	<0.05
Short-acting insulin analogue			
Total	0.30 ± 0.18	0.44 ± 0.17	0.001
T2DM	0.33 ± 0.13	0.50 ± 0.14	0.01
GDM	0.30 ± 0.19	0.38 ± 0.18	ns
Intermediate-acting insulin			
Total	0.12 ± 0.10	0.14 ± 0.07	ns
T2DM	0.18 ± 0.07	0.17 ± 0.07	ns
GDM	0.11 ± 0.09	0.11 ± 0.06	ns

T2DM: type 2 diabetes mellitus; GDM: gestational diabetes mellitus.
